# Environmental Distribution of AR Class 1 Integrons in Upper Adige River Catchment (Northern Italy)

**DOI:** 10.3390/ijerph17072336

**Published:** 2020-03-30

**Authors:** Federica Piergiacomo, Luigimaria Borruso, Sonia Ciccazzo, Stefano Rizzi, Stefan Zerbe, Lorenzo Brusetti

**Affiliations:** Faculty of Science and Technology, Free University of Bolzano-Bozen, Piazza Università 1, 39100 Bolzano-Bozen, Italy; federicapiergiacomo@gmail.com (F.P.); luigimaria.borruso@unibz.it (L.B.); cicchipower@gmail.com (S.C.); rizzi.s@alice.it (S.R.); Stefan.Zerbe@unibz.it (S.Z.)

**Keywords:** *intl1*, gene cassettes, antibiotic resistance, public health, bioindicators, land use

## Abstract

The source of antibiotic residuals can be directly related to the presence of municipal or industrial wastewater and agricultural activities. Antibiotics can trigger the dissemination of antibiotic resistance genes within bacterial communities. The mobile genetic elements Class 1 integrons (*intl1* region) has been already found to be correlated with a wide range of pollutants (i.e., antibiotics, heavy metals), and hence, it has been proposed as a proxy for environmental health. This study aimed to assess the presence of *intl1* in different environmental matrices, including agricultural and forest soils, freshwater and unpolluted sediments in the upper Adige River catchment (N Italy), in order to identify the spread of pollutants. *Intl1* was detected by direct PCR amplification at different frequencies. The urban and agricultural areas revealed the presence of *intl1*, except for apple orchards, where it was below the detection limit. Interestingly, *intl1* was found in a presumed unpolluted environment (glacier moraine), maybe because of the high concentration of metal ions in the mineral soil. Finally, *intl1* was absent in forest fresh-leaf litter samples and occurred with low rates in soil. Our results provide new data in supporting the use of *intl1* to detect the environmental health of different land-use systems.

## 1. Introduction

Antibiotics are naturally present in the environment, acting against bacterial infection or inhibiting their growth [1]. Antibiotics are produced by microorganisms as signal or regulation molecules, playing an important role in the interspecies competition too. Antibiotics are largely employed in human and animal medicine to prevent infections or to treat infectious diseases [2]. Despite the downward trend, Italy is still one of the European countries with the highest consumption of antibiotics per person, with 25.5 Defined Daily Dose (DDD) per 1000 Inhabitants Day (ID). Based on Trentino Alto Adige/South Tyrol, the average of regional consumption for the period 2013–2017 was equal to 17.0 DDD/1000 ID [3]. Concerning farming activities, in 2012 the European countries consumed 7982.0 tons of antibiotics for livestock production, whose 1534.3 tons were administered in Italy (third country after Germany and Spain) [4]. The application of manure as fertilizer may introduce antibiotics to land-use systems, and thus, increase antibiotic resistance in soil and water by enhancing phenomena, such as the spread of antibiotic-resistant genes (ARGs) in the environment. Considering urban wastewater, most of wastewater-treatment plants are not designed to remove antibiotics, and their effluent still contain them, thereby strongly affecting soil, water quality and possibly human health. The environmental concentration of ARGs could be increased, not only by antibiotics, but also by other pollutants, such as heavy metals [5].

Due to their important environmental functions, ARGs are easily spread into the microbiota by Horizontal Gene Transfer (HGT) [6]. Among the different mechanisms, one of the most important are integrons. Integrons are mobile genetic elements able to capture, excise and express genes’ cassettes, often encoding key factors, such as ARGs [7]. They are frequently embedded into the bacterial genome (~10% of bacteria) [8] or into plasmids in a wide variety of environments. Integrons are genetic elements, composed of three main parts, i.e., the intl gene, the *attI* recombination site, and the *Pc* integron associated promoter, located within *intl* and *attI* [9]. Class 1 integrons (*intl1*) are the most prevalent type of integrons found in clinical isolates, often hosting genes generally found in freshwater and soil Proteobacteria [7]. Their presence has been associated to different levels of pollution, acting as possible bioindicators and generic proxy of pollution [10]. The implementation of culture-independent techniques, such as PCR and metagenomics, for studying antibiotic resistance in the environment has resulted in the discovery of a high abundance and diversity of antibiotic resistance genes. Numerous studies have employed PCR for the detection of ARGs, highlighting its importance as a powerful tool of investigation, despite the methodological limitations e.g., *Taq* polymerases contamination [11]. Undoubtedly, there is an urgent need for advancements in tracking these elements in the environment and developing an overall view of resistance gene datasets, in order to develop appropriate approaches and standards for the detection of pollutants into the environment [12].

In our study, we investigated the presence of *intl1* occurring in bacterial communities through the direct amplification (PCR) of environmental DNA from channel sediments and soils/litter samples collected in Trentino Alto Adige/South Tyrol (Italy), using primers targeting the 5′-and 3′-conserved segments. Our approach aimed to chart the distribution of resistance selection in samples sourced from different land-use sites, i.e., urban, agricultural, forest and pristine environments.

## 2. Materials and Methods

### 2.1. Experimental Set-Up

In South Tyrol, we considered different environmental matrices from several land-use systems, including freshwater sediments as well as agricultural and forest soils. All the environmental samples were collected in the upper Adige River catchment in Northern Italy (Table 1; Figure S1). For all the sampling sites, a total of 169 samples in triplicates, were collected: (i) Channel sediments and rhizosphere samples were taken in environments characterized by agricultural activities (i.e., orchards or vineyards); (ii) channel sediment samples were collected from urban environments; (iii) soil and litter samples were picked up in managed forests, and from pristine post-glacial sediments.

### 2.2. Sampling Procedure

Samples were collected using sterile 50 mL tubes and immediately stored in a cool bag. Sampling procedures were applied, as shown by Borruso et al. [10] for sediments and by Esposito et al. [13] and other types of samples.

### 2.3. DNA Extraction and Quantification

From each tube, approximately 1.5 g of content for sediments and 0.4 g for soil samples was weighted. Total DNA was extracted by using PowerSoil^®^ DNA Isolation Kit (MoBio, Arcore, Italy) and quantified by using Qubit^®^ 2.0 Fluorometer Assay (Life Technologies Corporation). Concentration was adjusted to ≈ 10 ng/μl. Then, the extracted DNA was stored at −20 °C until its lab analysis.

### 2.4. PCR Amplifications

PCR amplifications were performed, as reported by Borruso et al. [10], by using a PTC-200 (Biorad, Milan, Italy) thermal cycler. DNA extracted from *Salmonella enterica* serovar Typhimurium 490 was used as positive control. Negative controls were included in the reactions to label any potential background contamination. PCR products were separated on electrophoresis agarose gel at 1.5% at 100 V for 7 h and visualized in a UV transilluminator (Versadoc Bio-Rad). The detection limit of the PCR was determined according to Borruso et al. [10]. Briefly, we employed serial dilutions of three biological replicates of *Salmonella enterica* serovar Typhimurium 490 cells containing a class 1 integron that harbored two gene cassettes (blaOXA-30, aadA1) as template DNA for the amplification. We used the dilution that showed amplification to estimate the minimum number of target genes necessary to produce a detectable band. The environmental samples that showed positive results from *intl1* gene amplification, were then amplified to determine the presence of the variable region according to the protocol of Borruso et al. [10]. The most suitable integron component for the detection is the *intl1* gene because of its homogeneity and conservation [14].

## 3. Results and Discussion

**Figure 1:**
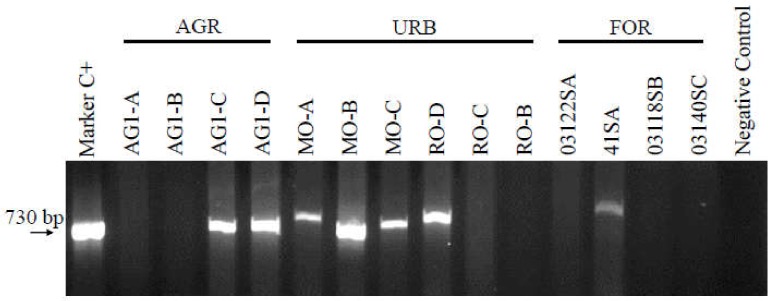
PCR amplification of agricultural (AGR/ AG1-A, AG1-B, AG1-C, AG1-D), urban (URB/ MO-A, MO-B, MO-C, RO-D, RO-C, RO-B) and forest samples (FOR/ 03122SA, 41SA, 03118SB, 03140SC) of Mori/Rovereto/Monticolo-Montiggl respectively, using *S. enterica*, as both size marker and positive control (Marker/C+ at 730 bp). Further information about each sample are available in Table S1, Table S2, Table S3, Table S4 of Supplementary Materials.

DNA samples from the sediments of urban, industrial, agricultural and non-polluted areas in the upper Adige River catchment of Trentino Alto Adige/South Tyrol, were examined for the presence of intl1 genes by PCR (Figure 1). We chose to investigate samples from sediments, as well as soils from the most representative land use systems of this region, ranging from freshwaters to moraines, forests and orchards. Although a very large territory, our approach covered the entire catchment with a considerable number of samples.

*Intl1* was not observed in the eDNA samples from the fresh-leaf litter, while it was found with different intensities in the agricultural, urban, forest and glacier moraine areas (Table 2). Our findings were mostly consistent with several studies, which already demonstrated the fate and the dynamics of class 1 integrons and ARGs in many environments, as well as their positive correlation with the site pollution level (Table S1). The novelty of our approach was to extend the use of intl1 as a proxy of environmental quality to a large territory, although with the awareness that intl1 may include different genes beside the resistance to antibiotics.

### 3.1. Intl1 Detection in Agricultural Channels and Apple Orchards

Specifically, for the agricultural area, four samples over the 10 collected in agricultural channels showed the presence of *intl1* at various intensities (e.g., higher in the channel Cameras, lower in the rivers Noce and Lusina). In general, at least one positive sample in all the investigated channels was found. These results confirm the presence of *intl1* in agricultural channels, indicating that the agricultural activity could have an influence in their diffusion by releasing wastewaters containing organic fertilizers, pesticides, and animal excrements (e.g., from cattle and sheep) which spread the presence of integrons [15,16]. In contrast, in samples collected in the rhizosphere of apple orchards, *intl1* was found at a lower frequency and intensity than in the channel samples. Moreover, in the experimental apple orchard of Caldaro-Kaltern the presence was not observed (0/35). The absence of detection in some orchards could be related to the use of mineral fertilizers rather than organic manure.

### 3.2. Intl1 Detection in Urban Channels

Findings for agricultural channel samples were like the ones observed for urban samples (6 positive samples over a total number of 14). Two samples, collected in a stream before and after the village of Mori, revealed an increase of *intl1* along the stream. In this specific case, some isolated houses are not linked to the wastewater pipeline, so that their effluents could affect the overall quality of the catching stream (Figure S1).

### 3.3. Intl1 Detection in Forest Soil, Leaf-Litter Samples and Post-Glacial Sediments

The presence of the *intl1* was also detected in forest samples. In leaf-litter samples, the results showed the complete absence of *intl1* (0/20) mostly lead to optimal conditions supplied by the rhizosphere for the bacterial growth. Under lower stress conditions the HGT is reduced and the transfer of integrons is inhibited [17]. Regarding the soil, for 6 samples, over 26 were positive and confirmed the presence of integrons. Considering the low human impact in the sampled forest, the presence of these signals could not be directly related to the presence of antibiotics, but probably to the relatively high concentration of heavy metals in the soil due to the acidic iron-rich porphyre. Indeed, Monticolo forest stands over a small hill of volcanic origin, and hence heavy metals are a major content of its soil chemistry. This could also explain the *intl1* retrieval in the Val di Mazia-Matschertal (4 positives over 36), a quite wild Alpine valley where human impact is limited to some sheep farming. The presence of *intl1* could be explained by the natural richness in metals of the Alpine moraine soils [17]. Heavy metals can lead to stress conditions for bacterial communities promoting a rapid HGT, to develop possibly favorable genes in their genomes in response to an adaptive ability [18,19].

### 3.4. Gene Cassettes Detection

Over the total amount of 169 samples, 29 were analyzed with the specific procedure previously described, for the cassette detection (Table 2). The presence of gene cassettes was observed in 25 samples, which showed different DNA band intensity and different patterns. Samples from the same sampling site showed similar or quite identical band patterns, and also similar DNA band intensity. This confirms that gene cassettes are specific for each environmental condition (and for each type of stress factor), and consequently that there is a specific population per each environmental matrix, as stated in other studies e.g., [9].

## 4. Conclusions

In conclusion, this study firstly shows how the presence of *intl1* is in the environment at different amount, according to different land uses in the upper Adige River catchment, reinforcing the hypothesis that *intl1* is one of the potential bioindicators of environmental quality, and health, respectively. In particular, *intl1* regions were discovered in higher quantity in samples from areas characterized by high human impact, such as agricultural and urban samples. We also found *intl1* in environments not characterized by human activities, but were present in very low amount compared to the human-impacted areas. This can be explained by other ecological factors, such as interspecific competition between bacteria in oligotrophic and metal-rich soils. The analysis of the gene cassette length showed that these genetic elements are present in our environments, forming specific populations per each situation associated with resistance to certain antibiotics and other polluting substances. In view of a renewed awareness within the One Health Approach applied to the open environment [20], this research shows the need to include several land use systems into the search of potential disseminating DNA regions, besides the urban-related ones. Further and detailed studies with a metagenomics approach will be fundamental, in order to achieve a better insight in the ecology of resistance, as well as the dynamics and the trends of bacterial responses to human pollution and set proper diagnostic strategies.

## Figures and Tables

**Table 1:** Name of sites, location, GPS coordinates, land use matrix and number of samples collected per each site.

Name	Site	Location	GPS	Land Use Matrix	N. of Samples
AGR1	Mori	Rio Cameras Channel	45°51′8.53″N 10°57′53.56″E	Agricultural sediments	4
MO	Mori	Rio Cameras Channel	45°51′17.43″N 10°59′12.57″E	Urban sediments	4
RO	Rovereto	Leno Stream	45°52′51.38″N 11° 1′20.54″E	Urban sediments	4
TRE	Trento	Fersina Stream	46°2′38.80″N 11° 7′6.39″E	Urban sediments	3
AGR2	Mezzolombardo	Noce River	46°13′8.19″N 11° 6′6.42″E	Agricultural sediments	3
AGR3	Ora-Auer	Lusina Channel	46°21′24.91″N 11°17′55.99″E	Agricultural sediments	3
BO	Bolzano	Talvera Stream	46°29′40.73″N 11°20′52.20″E	Urban sediments	3
CA	Caldaro-Kaltern	Apple orchard	46°21′18.44″N 11°16′36.81″E	Agricultural soil and rhizosphere	3
ME1	Sporminore	Apple orchard	46°14′13.38″N 11° 2′23.03″E	Agricultural soil	3
ME2	Sporminore	Apple orchard	46°14′12.63″N 11° 2′18.88″E	Agricultural soil	3
ME3	Ora-Auer	Apple orchard	46°21′29.65″N 11°18′3.79″E	Agricultural soil	3
ME4	Ora-Auer	Apple orchard	46°21′32.14″N 11°18′4.97″E	Agricultural soil	3
MON1	Monticolo-Montiggl	Oak forest	46°25′28.78″N 11°17′7.35″E	Oak rhizosphere	26
MON2	Monticolo-Montiggl	Oak forest	46°25′28.78″N 11°17′7.35″E	Fresh leaf litter	20
VAL	Val di Mazia-Matschertal	Glacier moraine	46°46′30.00″N 10°41′46.00″E	High mountain rhizosphere	36

**Table 2:** Land use, site, matrix, N. of samples ^1^ (number of analyzed samples) and N. of samples ^2^ (number of positive samples) and the number of bands and their intensity (*Intl1* region n. of bands and gene cassette n. of bandsranging from null to three detected bands) obtained by *intl1* and gene cassettes. (+ = low intensity; ++ = medium intensity; +++ = high intensity; n.a. = not applicable; - = not detected).

Land Use	Site	Matrix	N. of Samples ^1^	*Intl1* Region n. of Bands				N. of Samples ^2^	Gene Cassette n. of Bands			
				0	1	2	3		0	1	2	3
Vineyard	Mori	Sediments	4	2	2(++)	-	-	2	-	2(+)	-	-
Vineyard	Mezzolombardo	Sediments	3	2	1(++)	-	-	1	-	1(+)	-	-
Apple orchard	Ora-Auer	Sediments	3	2	1(+)	-	-	1	1	*-*	-	-
Apple orchard	Caldaro-Kaltern	Rhizosphere and soil	35	35	-	-	-	None	n.a.	n.a.	n.a.	n.a.
Apple orchard	Val di Non	Soil	6	3	2(+)	-	1(+)	None	n.a.	n.a.	n.a.	n.a.
Apple orchard	Ora-Auer	Soil	6	3	2(+)	2(+)	-	1	-	1(+)	-	-
Urban	Mori	Sediments	4	-	3(++); 1(+++)	-	-	4	-	-	4(++)	n.a.
Urban	Rovereto	Sediments	4	3	1(+)	-	-	1	1	-	-	-
Urban	Trento	Sediments	3	2	1(+)	-	-	1	1	*-*	-	-
Urban	Bolzano	Sediments	3	3	-	-	-	None	n.a.	n.a.	n.a.	n.a.
Forest	Monticolo-Montiggl	Rhizosphere	26	14	10(+)	2(+)	-	6	-	1(+); 1(++)	4(++)	-
Pristine	Val di Mazia-Matschertal	Mineral soil	36	32	4(+)	-	-	4	-	4(+)	-	-

^1^ Number of analyzed samples; ^2^ Number of positive samples at the *Intl1* detection.

## Supplementary Materials

The following are available online at https://www.mdpi.com/1660-4601/17/7/2336/s1, Figure S1: GIS map (1:900.000) of sampling sites (1-2: Mori/AGR1-MO, 3: Rovereto/RO, 4: Trento/TRE, 5: Mezzolombardo/ AGR2, 6: Ora-auer/AGR3, 7: Bolzano/ BO, 8: Caldaro-Kaltern/CA, 9-10: Sporminore/ME1-ME2, 11-12: Ora-auer/ME3-ME4, 13: Monticolo-Montiggl/MON1-MON2, 14: Val di Mazia-Matschertal/VAL) in Trentino-Alto Adige region; Table S1: main articles related to the dissemination of intl1 into non-clinical open environments. Table S2: *Intl1* PCR amplification results from samples collected from sediments within vineyard and apple orchard agricultural sites; Table S3: *Intl1* PCR amplification results from samples collected from grass rhizosphere, tree rhizosphere and root-free soil within vineyard and apple orchard agricultural sites; Table S4: *Intl1* PCR amplification results from samples collected from stream or river sediments within urban sites; Table S5: *Intl1* PCR amplification results from samples collected from soil and litter within the Monticolo-Montiggl forest site; Table S6: *Intl1* PCR amplification results from samples collected in mineral soils from Val di Mazia-Matschertal glacier moraine.
