# Supplementary material for: Environmental Distribution of AR Class 1 Integrons in Upper Adige River Catchment (Northern Italy)
Source: Int J Environ Res Public Health. 2020 Mar 30;17(7):2336. doi: 10.3390/ijerph17072336 (PMC7177501; doi:10.3390/ijerph17072336)
Supplement: Supplementary file 1 [file ijerph-17-02336-s001.pdf]

Environmental distribution of AR Class 1 integrons in upper Adige river catchment (Italy)

Federica Piergiacomo · Luigimaria Borruso · Stefano Rizzi · Stefan Zerbe · Lorenzo Brusetti

Faculty of Science and Technology, Free University of Bolzano-Bozen, Piazza Università 1, 39100 Bolzano-Bozen, Italy

1. Description of sampling sites

Trentino - Alto Adige cover different environments ranging from forests to urban centers, from vineyards and apple orchards to glaciers.

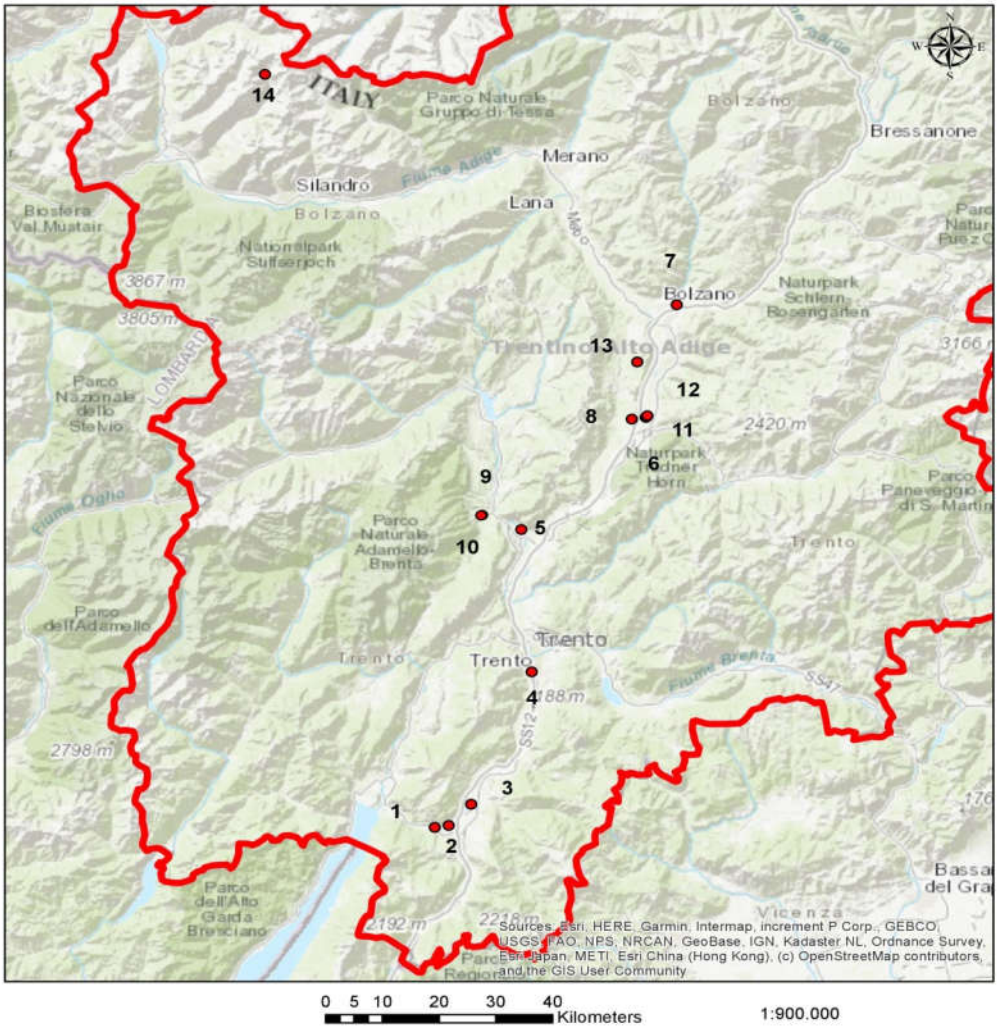

Figure S1. Gis map (1:900.000) of sampling sites (1-2:Mori/AGR1-MO, 3: Rovereto/RO, 4:Trento/TRE, 5: Mezzolombardo/ AGR2, 6: Ora-auer/AGR3, 7: Bolzano/ BO, 8: Caldaro-Kaltern/CA, 9-10: Sporminore/ME1-ME2, 11-12:Ora-auer/ME3-ME4, 13: Monticolo-Montiggl/MON1-MON2, 14: Val di Mazia-Matschertal/VAL) in Trentino-Alto Adige region.

1.1. Agricultural sampling sites:

1. Mori (AGR1): Sediments of a channel (Rio Cameras) that flows in the middle of an area characterized by vineyards. The samples were collected nearby a small cattle shed. The channel is natural, with the presence of grass and other spontaneous vegetation on the banks. The sediments are mainly composed by small size gravel and sand.

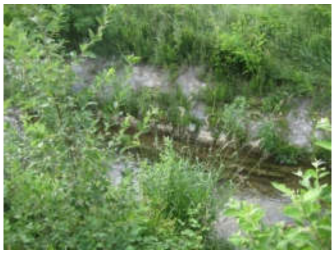

2. Mezzolombardo (AGR2): Sediments of a river (Noce) that flows in a valley (Val di Non) were apple orchards are enormously diffused. The sampling sites were at the end of the valley. This natural river shows banks covered by grass. The sediments are mainly formed by gravel.

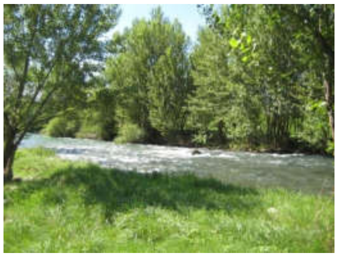

3. Ora-Auer (AGR3): Sediments of a channel (Rio Lusina) that flows through vineyards and apple orchards, at the Adige Valley bottom. This natural channel shows banks covered by grass and spontaneous vegetation. Sediments are mainly formed by sand.

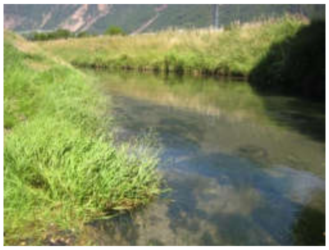

4. Sporminore (ME1/ME2): Soil samples collected in two apple orchards located in a valley (Val di Non) where the apple production represents the main activity. The soil samples were collected near the apple trees in a root-free zone.

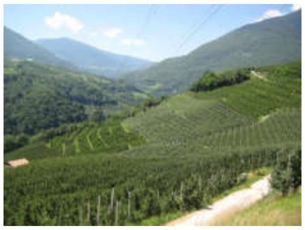

5. Ora-Auer (ME3/ME4): Soil samples were collected in two apple orchards located in the valley (Adige Valley). The soil samples were collected under the apple trees in a root-free zone.

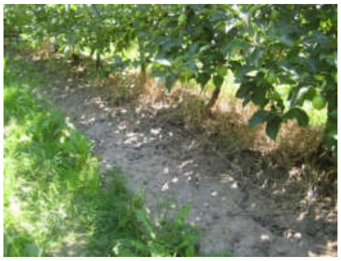

6. Caldaro-Kaltern (CA): Soil samples were collected in an experimental apple orchard, located in the valley (Adige Valley). The samples collected from tree rhizosphere, interrow grass rhizosphere and root-free soil. Orchards were planted in 2001 with trees of the cultivar Fuji (grafted on M9 rootstock) on a soil previously hosting another apple planting. The orchard is managed following the guidelines of organic farming, has a permanent grass cover in the alley (1.8 m large), while the soil area underneath the trees (1.2 m large) is kept free from weeds by periodic mechanical tillage

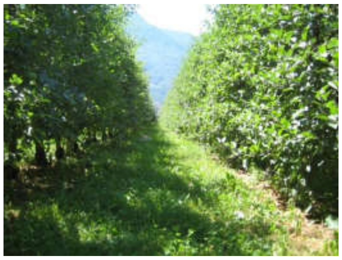

on the top soil layer. Each year approximately 600 Kg of an organic fertilizer (Nutristart) were applied to the soil-stripe underneath the trees. The soil is classified as loamy and the pH is 7.3.

1.2. Urban sampling sites:

1. Mori (MO): sediments of a channel (Rio Cameras) that flows through the town of Mori (approximately 10,000 inhabitants). The sampling site was located at the end of the urban centre. The effluent of a small wastewater treatment plant, collecting wastewater from small villages located near Mori, have been known to be discharged into the Rio Cameras. The channel presents artificial banks, and the sediments were mostly sandy.

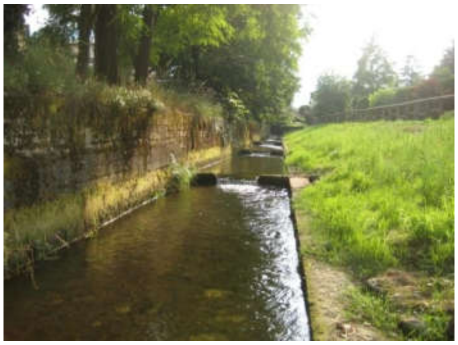

2. Rovereto (RO): Sediments of a stream (Leno) that flows inside the town of Rovereto (approximately 40,000 inhabitants). The sampling site was located at the end of the urban centre. A hospital is located at an approximate distance of 250 m from the stream. The banks are mainly artificial, with some areas covered by vegetation. Sediments mainly composed by gravel.

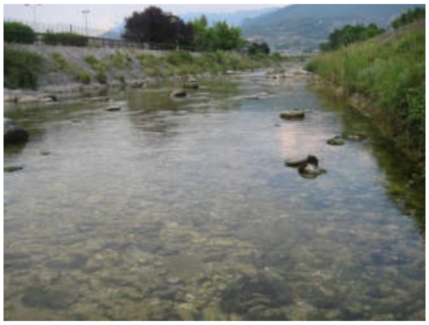

3. Trento (TRE): Sediments of a stream (Fersina) that crosses the city of Trento (approximately 100,000 inhabitants). The stream flows 400 meters from the city hospital. The sampling site was located immediately before the confluence between Fersina and Adige River, at the end of the city. Banks are mainly artificial, with some areas covered by vegetation. Sediment is composed by gravel and sand.

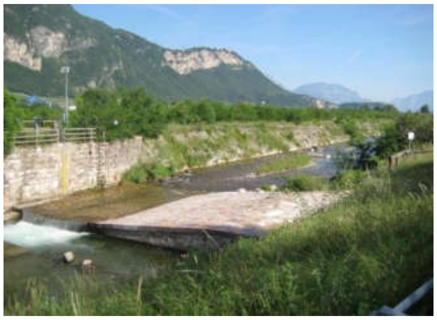

4. Bolzano (BO): Sediments of a stream (Talvera) that flows in the middle of the city (approximately 100,000 inhabitants). Banks are natural, and the sediment is sandy.

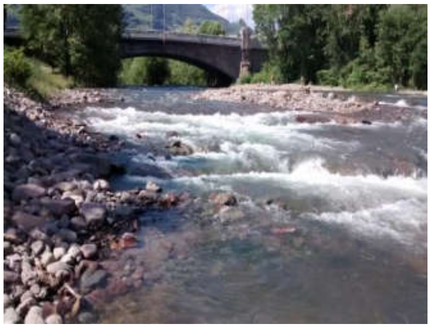

1.3. Forest sampling site:

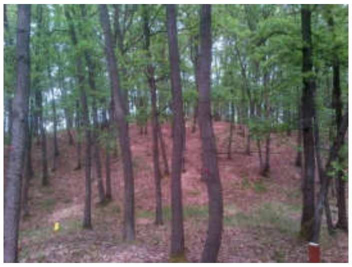

1. Monticolo-Montiggl (MON1-MON2): Oak rhizosphere samples collected in different sites inside a managed forest nearby the lake of Monticolo. The sampling area was characterized by the presence of sessile oak (*Quercus petraea* Liebl). In addition fresh litter gathered during the fall season were analyzed.

<u>Glacial sediments:</u>

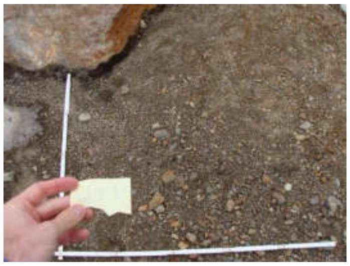

1. Val di Mazia-Matschertal (VAL): Mineral soil samples collected in a post glacial environment at 2,750 m a.s.l. The mineral soil was ice-free since 50 years.

2. Results and Discussion

Table S1. Environmental studies investigating the correlation between class 1 integrons, antibiotic resistance genes and pollution (Year, References, Location, Detected genes in the study, Analysis used, Type of sample collected, Land use system of the investigated site and Findings showed). Citations references are reported below the table.

Year	References	Location	Detected genes	Analysis	Type of sample	Land use system	Findings
2017	Gillings [1]	worldwide	class 1 integrons	review	not applicable	not applicable	Class 1 integrons are spread via the selection pressures and pollutants arising out of human activity.
2017	Zhu et al. [2]	China	248 ARGs, 9 transposase genes, intl1, 16S rRNA	HT-qPCR	sediment	estuarine wetlands	Human activity influences the abundance and dissemination of ARGs
2016	Koczura et al. [3]	Poland	intl1, sul1, sul2	qPCR	water, sediment	river, wwtp effluent discharge	The abundance of class 1 integrons in aquatic habitat is due to anthropogenic pressure and environmental factors
2016	Borruso et al. [4]	China	intl1, sul1	PCR	sediment	freshwater habitat	Intl1 and gene cassettes depend on the pollution gradient
2015	Chen et al. [5]	South China	int1, sul1, sul2, sul3, qnrS, ermB	PCR, qPCR	water, sediment	river catchment, estuary	Integrons foster the dissemination of ARGs in human-impacted aquatic environments.
2014	Gillings et al. [6]	worldwide	class 1 integrons	review	not applicable	not applicable	The relationship between anthropogenic impacts and the abundance of intI1 is examined
20	Dealtry et al. [7]	Belgium	IncP-1 group, sul1, sul2, intl1, intl2, aadA	PCR, Southern blot	biopurification system mix	farm system	Correlation between IncP plasmid abundance and pesticide impact

14				hybridization			
2014	Jechalke et al. [8]	Germany	intl1, intl2, qacE, qacEΔ 1	PCR, Southern blot hybridization	manure-treated/untreated soils, rhizosphere, digestates, non-farm biopurification system	farm manuring	Intl1 and other genes increase their abundance
2013	Cheng et al. [9]	China	10 tet genes, sul1, sul2, intl1	qPCR	animal manures and wastewater lagoon samples	manure, wastewater	Intl1 and other genes increase their abundance due to animal manure
2013	Khan et al. [10]	Pakistan	intl1, tetA, sul1, dfrA1, ermB	qPCR	water	river catchment	Human impact increases Intl1 and other gene
2012	Drudge et al. [11]	Canada	aac(3)Ia, aadA1, sul2, tetA, tetC	microarray	water, floc	freshwater habitat	Human impact increases Intl1 cassettes
2011	Heuer et al. [12]	worldwide	class 1 integrons	review	different animal manure stocks	animal husbandry	Agricultural antibiotic use has a significant effect on the spread of resistance in the human community
2011	La Para et al. [13]	USA	tet(A), tet(X), tet(W), intI1, 16S rRNA	qPCR	Water, sediment	wastewater treatment	The effluent shows a significative Intl1 abundance increase
2010	Storteboom et al. [14]	USA	tet(B), tet(C), tet(E), tet(H), tet(M), tet(O), tetB(P), tet(Q), tet(S), tet(T), tet(W), sul(I), sul(II)	PCR, RFLP	water	river	The transport of ARGs from specific sources is likely the dominant mechanism for ARG proliferation
2010	Byrne-Bailey et al. [15]	Not specified	intll, intI2	qPCR	amended soil	farm manuring	Pig slurry-amended soil is a reservoir of diverse bacterial species carrying class 1 and 2 integrons
20	McKinney et al. [16]	USA	tet(O), tet(W), sul(I),sul(II)	qPCR	water	livestock lagune	The fate of ARGs in lagoons used to treat animal waste is investigated

10							
2009	Holzel et al. [17]	Germany	tet(M), tet(O), tet(B), SULs	qPCR	liquid pig manure	animal husbandry	Antibiotic contamination of manure seems to be associated with a variety of changes in bacterial resistance
2009	Binh et al. [18]	Germany	aadA1, aadA2, aadA9, aadA11, aadA13	PCR, cloning, RFLP, sequencing	manured/not manured soil, manure	soil fertilization	aadA genes localized on class 1 integrons are introduced via piggery manure into agricultural soils
2009	Zhang et al. [19]	China	intl1, tetA, tetC	PCR, qPCR	water, sediment	wastewater treatment	Intl1 grows in abundance downstream from city
2009	Martinez et al. [20]	worldwide	antibiotic resistance genes	review	review	review	Shows the resistance pathogen acquisition system and the consequences in the environment
2008	Hardwick et al. [21]	Australia	intl1, 16S rRNA	qPCR	sediment	stream chatchment	Human impact promotes Intl1 abundance
2008	Binh et al. [22]	Germany	81 plasmids, bla-TEM, sul1, sul2, sul3	PCR, qPCR, hybridization	animal manures	manure storage tanks	Piggery manure is a reservoir of broad-host range plasmids conferring multiple antibiotic resistance genes
2008	Byrne-Bailey et al. [23]	UK	sul1, sul2, sul3, intl1, intl2, qacE, qacEΔ 1	PCR, sequencing	manured soils, slurry	farm manuring	Sulfonamide resistance in *Psychrobacter*, *Enterococcus*, and *Bacillus* spp. is reported
2007	Heuer et al. [24]	Not specified	sul1, sul2, sul3	PCR, hybridization	animal manure, manured soil with and without the addition of sulfadiazine	soil microcosms	Manure from treated pigs enhances the spread of antibiotic resistances in soil bacterial communities
2007	Peak et al. [25]	USA	tet(O), tet(Q), tet(W), tet(M), tet(B), tet(L)	qPCR	water	feedlot lagune	Antibiotics influence the abundance and seasonal distribution of resistance genes in associated lagoons

2007	Ghosh and la Para [26]	USA	tet(A), tet(B), tet(C), tet(D), tet(E), tet(G), tet(K), tet(L), tetA(P), tet(M), tet(O), tet(S), tet(Q), tet(X)	Multiplex PCR	soil	farm manuring	Excessive application of animal manure leads to the spread of resistance to soil bacteria
2007	Koike et al. [27]	Not specified	tet(M), tet(O), tet(Q), tet(W), tet(C), tet(H),tet(Z)	qPCR	water	lagoon, groundwater, swine manuring	Antibiotic resistance genes in groundwater depended on swine manure, but they are also part of the indigenous gene pool
2007	Boucher et al. [28]	worldwide	integrons	review	not applicable	not applicable	The diversity and the evolution of integrons are showed

1. Gillings, M. R. Class 1 integrons as invasive species. *Curr. Opin. Microbiol*. **2017**, *38*, 10-15.
2. Zhu, Y.G.; Zhao, Y.,; Li, B.; Huang, C.L.,; Zhang, S. Y.; Yu, S.; et al. Continental-scale pollution of estuaries with antibiotic resistance genes. *Nat. Microbiol*. **2017**, *2*(4), 16270.
3. Koczura, R.; Mokracka, J.; Taraszewska, A.; Łopacinska, N. Abundance of class 1 integron-integrase and sulfonamide resistance genes in river water and sediment is affected by anthropogenic pressure and environmental factors. *Microb. Ecol*. **2016**, *72*(4), 909-916.
4. Borruso, L.; Harms, K.; Johnsen, P.J.; Nielsen; K.M.; Brusetti, L. Distribution of class 1 integrons in a highly impacted catchment. *Sci. Total Environ*. **2016**, 566, 1588-1594
5. Chen, B.; Liang, X. ; Nie, X. ; Huang, X. ;Zou, S. ; Li, X. The role of class I integrons in the dissemination of sulfonamide resistance genes in the Pearl River and Pearl River Estuary, South China. *J. Hazard. Mater*. **2015**, *282*, 61-67.
6. Gillings, M.R. Integrons: Past, Present, and Future. *Microbiol. Mol. Biol. Rev*. **2014**, 78, 257–277
7. Dealtry, S.; Holmsgaard, P.N.; Dunon, V.; Jechalke, S.; Ding, G.C.; Krögerrecklenfort, E.; Smalla, K. Shifts in abundance and diversity of mobile genetic elements after the introduction of diverse pesticides into an on-farm biopurification system over the course of a year. *Appl. Env. Microb.* **2014**, 80, 4012–4020
8. Jechalke, S.; Schreiter, S.; Wolters, B; Dealtry, S.; Heuer, H.; Smalla, K. Widespread dissemination of class 1 integron components in soils and related ecosystems as revealed by cultivation-independent analysis. *Front. Microbiol*. **2014**, 4, 420.
9. Cheng, W.; Chen, H.; Su, C.; Yan, S. Abundance and persistence of antibiotic resistance genes in livestock farms: A comprehensive investigation in Eastern China. *Environ. Int.* **2013**, 61, 1–7
10. Khan, G.A.; Berglund, B.; Khan, K.M.; Lindgren, P-E.; Fick, J. Occurrence and abundance of antibiotics and resistance genes in rivers, canal and near drug formulation facilities—a study in Pakistan. *PLoS One.* **2013**, 8, e62712.
11. Drudge, C.N.; Elliott, A.V.C.; Plach, J.M.; Ejim, L.J.; Wright, G.D.; Droppo, I.G. et al. Diversity of integron- and culture-associated antibiotic resistance genes in freshwater floc. *Appl. Environ. Microbiol.* **2012**, 78,4367–4372.
12. Heuer, H.; Schmitt, H. ; Smalla, K. Antibiotic resistance gene spread due to manure application on agricultural fields. *Curr. Opin. Microbiol*. **2011**, 14(3), 236-243.
13. LaPara ;T.M.; Burch, T.R.; McNamara, P.J.; Tan, D.T.; Yan, M.; Eichmiller, J.J. Tertiary-treated municipal wastewater is a significant point source of antibiotic resistance genes into Duluth-Superior Harbor. *Environ. Sci. Technol.* **2011**, 45,9543–9549.
14. Storteboom, H.; Arabi, M.; Davis, J. G.; Crimi, B.; Pruden, A. Tracking antibiotic resistance genes in the South Platte River basin using molecular signatures of urban, agricultural, and pristine sources. *Environ. Sci. Technol*. **2010**, *44*(19), 7397-7404.

15. Byrne-Bailey, K. G.; Gaze, W. H.; Zhang, L.; Kay, P; Boxall, A.; Hawkey, P. M.; Wellington, E. M. H. Integron prevalence and diversity in manured soil. *Appl. Environ. Microbiol.* **2011**, 77(2), 684-687.
16. McKinney, C. W., Loftin, K. A., Meyer, M. T., Davis, J. G., & Pruden, A. Tet and sul antibiotic resistance genes in livestock lagoons of various operation type, configuration, and antibiotic occurrence. *Environ. Sci. Technol.* **2010**, *44*(16), 6102-6109.
17. Hölzel, C. S.; Harms, K. S.; Küchenhoff, H.; Kunz, A.; Müller, C.; Meyer, K. et al. Phenotypic and genotypic bacterial antimicrobial resistance in liquid pig manure is variously associated with contents of tetracyclines and sulfonamides. *J. Appl. Microbiol*. **2010**, *108*(5), 1642-1656.
18. Binh, C. T.; Heuer, H. ; Kaupenjohann, M. ; Smalla, K. Diverse aadA gene cassettes on class 1 integrons introduced into soil via spread manure. *Res. Microbiol*. **2009**, *160*(6), 427-433.
19. Zhang, X.; Wu, B.; Zhang, Y.; Zhang, T.; Yang, L., Fang, H. et al. Class 1 integronase gene and tetracycline resistance genes *tetA* and *tetC* in different water environments of Jiangsu Province, China. *Ecotoxicol*. **2009**,18, 652–660.
20. Martinez, J. L. The role of natural environments in the evolution of resistance traits in pathogenic bacteria. *P Roy. Soc. B-Biol. Sci.* **2009**, 276(1667), 2521-2530.
21. Hardwick, S.A.; Stokes, H.W.; Findlay, S.; Taylor, M.; Gillings, M.R . Quantification of class 1 integron abundance in natural environments using real-time quantitative PCR. *FEMS Microbiol. Lett*. **2008**, 278, 207–212.
22. Binh, C. T. T.; Heuer, H.; Kaupenjohann, M.; Smalla, K. Piggery manure used for soil fertilization is a reservoir for transferable antibiotic resistance plasmids. *FEMS Microbiol. Ecol.* **2008**, 66(1), 25-37.
23. Byrne-Bailey, K. G.; Gaze, W. H.; Kay, P.; Boxall, A. B. A.; Hawkey, P. M. ; Wellington, E. M. H. Prevalence of sulfonamide resistance genes in bacterial isolates from manured agricultural soils and pig slurry in the United Kingdom. *Antimicrob. Agents Chemother*. **2009**, 53(2), 696-702.
24. Heuer, H.; Smalla, K. Manure and sulfadiazine synergistically increased bacterial antibiotic resistance in soil over at least two months. *Environ. Microbiol.* **2007**, 9(3), 657-666.
25. Peak, N.; Knapp, C. W.; Yang, R. K.; Hanfelt, M. M.; Smith, M. S.; Aga, D. S. et al. Abundance of six tetracycline resistance genes in wastewater lagoons at cattle feedlots with different antibiotic use strategies. *Environ. Microbiol*. **2007**, 9(1), 143-151.
26. Koike, S.; Krapac, I. G.; Oliver, H. D.; Yannarell, A. C.; Chee-Sanford, J. C.; Aminov, R. I. et al. Monitoring and Source Tracking of Production Facilities over a 3-Year Period and Groundwater Adjacent to Swine Tetracycline Resistance Genes in Lagoons. *Appl. Environ. Microbiol.* **2007**, 73(15):4813.
27. Ghosh, S.; LaPara, T. M. The effects of subtherapeutic antibiotic use in farm animals on the proliferation and persistence of antibiotic resistance among soil bacteria. The ISME J. 2007, 1(3), 191-203.
28. Boucher, Y.; Labbate, M.; Koenig, J.E.; Stokes, H.W. Integrons: mobilizable platforms that promote genetic diversity in bacteria. *Trends Microbiol*. **2007**, 15, 301–309

Supplementary results

Table S2. *Intl1* PCR amplification results from samples collected from sediments within vineyard and apple orchard agricultural sites. Site, Matrix, *Inlt1* number of bands and their intensity (Intl1 n. of bands -0 bands, 1 band, 2 bands, 3 bands) obtained. (+ = low intensity; ++ = medium intensity; +++ = high intensity; n.a. = not applicable; - = not detected).

**Site**	**Matrix**	**Sample name**	***Intl1* N. of bands**				
			**0**	**1**	**2**	**3**	**4**
Mori (Channel Cameras)	sediments	AG1 A	1	-	-	-	-
Mori (Channel Cameras)	sediments	AG1 B	1	-	-	-	-
Mori (Channel Cameras)	sediments	AG1 C	-	1(++)	-	-	-
Mori (Channel Cameras)	sediments	AG1 D	-	1(++)	-	-	-
Mezzolombardo (River Noce)	sediments	AG2 A	-	1(+)	-	-	-
Mezzolombardo (River Noce)	sediments	AG2 B	1	-	-	-	-
Mezzolombardo (River Noce)	sediments	AG2 C	1	-	-	-	-
Ora - Auer (Channel Lusina)	sediments	AG3 A	1	-	-	-	-
Ora - Auer (Channel Lusina)	sediments	AG3 B	-	1(+)	-	-	-
Ora - Auer (Channel Lusina)	sediments	AG3 C	1	-	-	-	-

Table S3. *Intl1* PCR amplification results from samples collected from grass rhizosphere, tree rhizosphere and root-free soil within vineyard and apple orchard agricultural sites. Site, Matrix, *Inlt1* number of bands and their intensity (Intl1 n. of bands -0 bands, 1 band, 2 bands, 3 bands) obtained. (+ = low intensity; ++ = medium intensity; +++ = high intensity; n.a. = not applicable; - = not detected).

Site	Matrix	Sample	*Intl1* N. of bands				
			0	1	2	3	4
Caldaro - Kaltern	Grass Rhizosphere	2G2 ka112	1	-	-	-	-
Caldaro - Kaltern	Grass Rhizosphere	4G1	1	-	-	-	-
Caldaro - Kaltern	Soil	3S2 ka112	1	-	-	-	-
Caldaro - Kaltern	Soil	1S1 ka112	1	-	-	-	-
Caldaro - Kaltern	Soil	2S1	1	-	-	-	-
Caldaro - Kaltern	Tree Rhizosphere	2T2 ka112	1	-	-	-	-
Caldaro - Kaltern	Soil	2S2 ka112	1	-	-	-	-
Caldaro - Kaltern	Tree Rhizosphere	3T2 ka113	1	-	-	-	-
Caldaro - Kaltern	Grass Rhizosphere	5G2 ka112	1	-	-	-	-
Caldaro - Kaltern	Soil	6S1 ka112	1	-	-	-	-
Caldaro - Kaltern	Soil	4S2 ka112	1	-	-	-	-
Caldaro - Kaltern	Tree Rhizosphere	1T1 ka112	1	-	-	-	-
Caldaro - Kaltern	Grass Rhizosphere	4G2 ka111	1	-	-	-	-
Caldaro - Kaltern	Tree Rhizosphere	3T2 ka112	1	-	-	-	-
Caldaro - Kaltern	Soil	6S1 ka113	1	-	-	-	-
Caldaro - Kaltern	Grass Rhizosphere	4G1 ka112	1	-	-	-	-
Caldaro – Kaltern	Grass Rhizosphere	6G2	1	-	-	-	-
Caldaro - Kaltern	Grass Rhizosphere	3G2	1	-	-	-	-
Caldaro - Kaltern	Tree Rhizosphere	2T1 ka112	1	-	-	-	-
Caldaro - Kaltern	Grass Rhizosphere	6G1 ka113	1	-	-	-	-
Caldaro - Kaltern	Soil	2S1 ka112	1	-	-	-	-
Caldaro - Kaltern	Grass Rhizosphere	1G2 ka111	1	-	-	-	-
Caldaro - Kaltern	Grass Rhizosphere	6G2 ka111	1	-	-	-	-
Caldaro - Kaltern	Grass Rhizosphere	4G2 ka113	1	-	-	-	-
Caldaro – Kaltern	Grass Rhizosphere	4G1 ka113	1	-	-	-	-
Caldaro - Kaltern	Grass Rhizosphere	5G2 ka111	1	-	-	-	-
Caldaro - Kaltern	Grass Rhizosphere	2G1 ka111	1	-	-	-	-
Caldaro - Kaltern	Soil	5S1 ka113	1	-	-	-	-
Caldaro - Kaltern	Grass Rhizosphere	3G1 ka111	1	-	-	-	-
Caldaro - Kaltern	Grass Rhizosphere	3G1 ka113	1	-	-	-	-
Caldaro - Kaltern	Tree Rhizosphere	4T2 ka113	1	-	-	-	-
Caldaro - Kaltern	Grass Rhizosphere	2G2 ka111	1	-	-	-	-
Caldaro - Kaltern	Grass Rhizosphere	5G1 ka111	1	-	-	-	-
Caldaro - Kaltern	Soil	4S1 ka111	1	-	-	-	-
Caldaro - Kaltern	Tree Rhizosphere	6T2 ka113	1	-	-	-	-
Val di Non	Soil	ME1 A	-	1	-	-	-
Val di Non	Soil	ME1 B	-	1(+)	-	-	-
Val di Non	Soil	ME1 C	1	-	-	-	-
Val di Non	Soil	ME2 A	-	-	-	1(+)	-
Val di Non	Soil	ME2 B	1	-	-	-	-
Val di Non	Soil	ME2 C	1	-	-	-	-
Ora - Auer	Soil	ME3 A	-	1(+)	-	-	-
Ora - Auer	Soil	ME3 B	-	1	-	-	-
Ora - Auer	Soil	ME3 C	-	-	1(+)	-	-
Ora - Auer	Soil	ME4 A	1	-	-	-	-
Ora - Auer	Soil	ME4 B	1	-	-	-	-
Ora - Auer	Soil	ME4 C	1	-	-	-	-

Table S4. *Intl1* PCR amplification results from samples collected from stream or river sediments within urban sites. Site, Matrix, *Inlt1* number of bands and their intensity (Intl1 n. of bands -0 bands, 1 band, 2 bands, 3 bands) obtained. (+ = low intensity; ++ = medium intensity; +++ = high intensity; n.a. = not applicable; - = not detected).

Site	Matrix	Sample	*Intl1* N. of bands				
			0	1	2	3	4
**Mori (Channel Cameras)**	sediments	MO A	-	1(++)	-	-	-
**Mori (Channel Cameras)**	sediments	MO B	-	1(+++)	-	-	-
**Mori (Channel Cameras)**	sediments	MO C	-	1(++)	-	-	-
**Mori (Channel Cameras)**	sediments	MO D	-	1(++)	-	-	-
**Rovereto (River Leno)**	sediments	RO A	1	-	-	-	-
**Rovereto (River Leno)**	sediments	RO B	1	-	-	-	-
**Rovereto (River Leno)**	sediments	RO C	1	-	-	-	-
**Rovereto (River Leno)**	sediments	RO D	-	1(+)	-	-	-
**Trento (River Fersina)**	sediments	TR A	1	-	-	-	-
**Trento (River Fersina)**	sediments	TR B	1	-	-	-	-
**Trento (River Fersina)**	sediments	TR C	-	1(+)	-	-	-
**Bolzano (River Talvera)**	sediments	BZ A	1	-	-	-	-
**Bolzano (River Talvera)**	sediments	BZ B	1	-	-	-	-
**Bolzano (River Talvera)**	sediments	BZ C	-	-	-	-	-

Table S5. *Intl1* PCR amplification results from samples collected from soil and litter within the Monticolo-Montiggl forest site. Site, Matrix, *Inlt1* number of bands and their intensity (Intl1 n. of bands -0 bands, 1 band, 2 bands, 3 bands) obtained. (+ = low intensity; ++ = medium intensity; +++ = high intensity; n.a. = not applicable; - = not detected).

Site	Matrix	Sample	*Intl1* N. of bands				
			0	1	2	3	4
Monticolo-Montiggl	Rhizosphere	41SA	-	1(+)	-	-	-
Monticolo-Montiggl	Rhizosphere	03140NEB	1	-	-	-	-
Monticolo-Montiggl	Rhizosphere	03140SC	1	-	-	-	-
Monticolo-Montiggl	Rhizosphere	03122NEA	1	-	-	-	-
Monticolo-Montiggl	Rhizosphere	03122NEC	1	-	-	-	-
Monticolo-Montiggl	Rhizosphere	03122SC	-	1	-	-	-
Monticolo-Montiggl	Rhizosphere	03140 SA	-	1	-	-	-
Monticolo-Montiggl	Rhizosphere	03140NOA	-	1	-	-	-
Monticolo-Montiggl	Rhizosphere	03122NOA	-	1	-	-	-
Monticolo-Montiggl	Rhizosphere	03122SB	-	1	-	-	-
Monticolo-Montiggl	Rhizosphere	3155SA	-	1(+)	-	-	-
Monticolo-Montiggl	Rhizosphere	03155NEB	-	1	-	-	-
Monticolo-Montiggl	Rhizosphere	03118NOB	-	-	1(+)	-	-
Monticolo-Montiggl	Rhizosphere	03118NOA	-	1(+)	-	-	-
Monticolo-Montiggl	Rhizosphere	03118NEB	-	1(+)	-	-	-
Monticolo-Montiggl	Rhizosphere	03122SA	1	-	-	-	-
Monticolo-Montiggl	Rhizosphere	03122NEB	1	-	-	-	-
Monticolo-Montiggl	Rhizosphere	03122NOC	1	-	-	-	-
Monticolo-Montiggl	Rhizosphere	03140NEC	1	-	-	-	-
Monticolo-Montiggl	Rhizosphere	03118NEA	1	-	-	-	-
Monticolo-Montiggl	Rhizosphere	03141NOB	1	-	-	-	-
Monticolo-Montiggl	Rhizosphere	03122NOB	1	-	-	-	-
Monticolo-Montiggl	Rhizosphere	03118SB	1	-	-	-	-
Monticolo-Montiggl	Rhizosphere	03141NOA	-	-	1(+)	-	-
Monticolo-Montiggl	Rhizosphere	03140NEA	1	-	-	-	-
Monticolo-Montiggl	Rhizosphere	03140NOB	1	-	-	-	-

Table S6. *Intl1* PCR amplification results from samples collected in mineral soils from Val di Mazia-Matschertal glacier moraine. Site, Matrix, *Inlt1* number of bands and their intensity (Intl1 n. of bands -0 bands, 1 band, 2 bands, 3 bands) obtained. (+ = low intensity; ++ = medium intensity; +++ = high intensity; n.a. = not applicable; - = not detected).

Site	Matrix	Sample	*Intl1* N. of bands				
			**0**	**1**	**2**	**3**	**4**
**Val di Mazia-Matschertal**	Mineral Soil	63	1	-	-	-	-
**Val di Mazia-Matschertal**	Mineral Soil	109	1	-	-	-	-
**Val di Mazia-Matschertal**	Mineral Soil	60	1	-	-	-	-
**Val di Mazia-Matschertal**	Mineral Soil	64	1	-	-	-	-
**Val di Mazia-Matschertal**	Mineral Soil	20	1	-	-	-	-
**Val di Mazia-Matschertal**	Mineral Soil	105	1	-	-	-	-
**Val di Mazia-Matschertal**	Mineral Soil	148	1	-	-	-	-
**Val di Mazia-Matschertal**	Mineral Soil	151	1	-	-	-	-
**Val di Mazia-Matschertal**	Mineral Soil	53	-	1(+)	-	-	-
**Val di Mazia-Matschertal**	Mineral Soil	153	1	-	-	-	-
**Val di Mazia-Matschertal**	Mineral Soil	156	1	-	-	-	-
**Val di Mazia-Matschertal**	Mineral Soil	159	1	-	-	-	-
**Val di Mazia-Matschertal**	Mineral Soil	166	1	-	-	-	-
**Val di Mazia-Matschertal**	Mineral Soil	168	1	-	-	-	-
**Val di Mazia-Matschertal**	Mineral Soil	172	1	-	-	-	-
**Val di Mazia-Matschertal**	Mineral Soil	175	1	-	-	-	-
**Val di Mazia-Matschertal**	Mineral Soil	173	1	-	-	-	-
**Val di Mazia-Matschertal**	Mineral Soil	23	1	-	-	-	-
**Val di Mazia-Matschertal**	Mineral Soil	T0 95C	1	-	-	-	-
**Val di Mazia-Matschertal**	Mineral Soil	T0 89B	1	-	-	-	-
**Val di Mazia-Matschertal**	Mineral Soil	T0 90C	1	-	-	-	-
**Val di Mazia-Matschertal**	Mineral Soil	PS101DJ	-	1(+)	-	-	-
**Val di Mazia-Matschertal**	Mineral Soil	PS105DJ	-	1(+)	-	-	-
**Val di Mazia-Matschertal**	Mineral Soil	PS103DJ	-	1(+)	-	-	-
**Val di Mazia-Matschertal**	Mineral Soil	PS201DJ	1	-	-	-	-
**Val di Mazia-Matschertal**	Mineral Soil	PS202DJ	1	-	-	-	-
**Val di Mazia-Matschertal**	Mineral Soil	PS205DJ	1	-	-	-	-
**Val di Mazia-Matschertal**	Mineral Soil	PS201DO	1	-	-	-	-
**Val di Mazia-Matschertal**	Mineral Soil	PS202DO	1	-	-	-	-
**Val di Mazia-Matschertal**	Mineral Soil	PS205DO	1	-	-	-	-
**Val di Mazia-Matschertal**	Mineral Soil	PS303DJ	1	-	-	-	-
**Val di Mazia-Matschertal**	Mineral Soil	PS305DJ	1	-	-	-	-
**Val di Mazia-Matschertal**	Mineral Soil	PS306DJ	1	-	-	-	-
**Val di Mazia-Matschertal**	Mineral Soil	PS303DO	1	-	-	-	-
**Val di Mazia-Matschertal**	Mineral Soil	PS305DO	1	-	-	-	-
**Val di Mazia-Matschertal**	Mineral Soil	PS306DO	1	-	-	-	-
